# Supplementary material for: Structure of an MHC I–tapasin–ERp57 editing complex defines chaperone promiscuity
Source: Nat Commun. 2022 Sep 14;13:5383. doi: 10.1038/s41467-022-32841-9 (PMC9474470; doi:10.1038/s41467-022-32841-9)
Supplement: Supplementary file 1 — Supplementary Information [file 41467_2022_32841_MOESM1_ESM.pdf]

Supplementary Information

Structure of an MHC I–tapasin–ERp57 editing complex defines chaperone promiscuity

Ines Katharina Müller[1], Christian Winter[1], Christoph Thomas[1], Robbert M. Spaapen[2,3], Simon Trowitzsch[1]*, Robert Tampé[1]*

[1]Institute of Biochemistry, Biocenter, Goethe University Frankfurt; Max-von-Laue Strasse 9, 60438 Frankfurt/Main, Germany.

[2]Department of Immunopathology, Sanquin Research, Amsterdam, The Netherlands.

[3]Landsteiner Laboratory, Amsterdam UMC, University of Amsterdam, Amsterdam, The Netherlands.

*Corresponding authors. Email: trowitzsch@biochem.uni-frankfurt.de (ST);
Email: tampe@em.uni-frankfurt.de (RT)

Supplementary Figure 1-8
Supplementary Table 1

SUPPLEMENTARY FIGURES

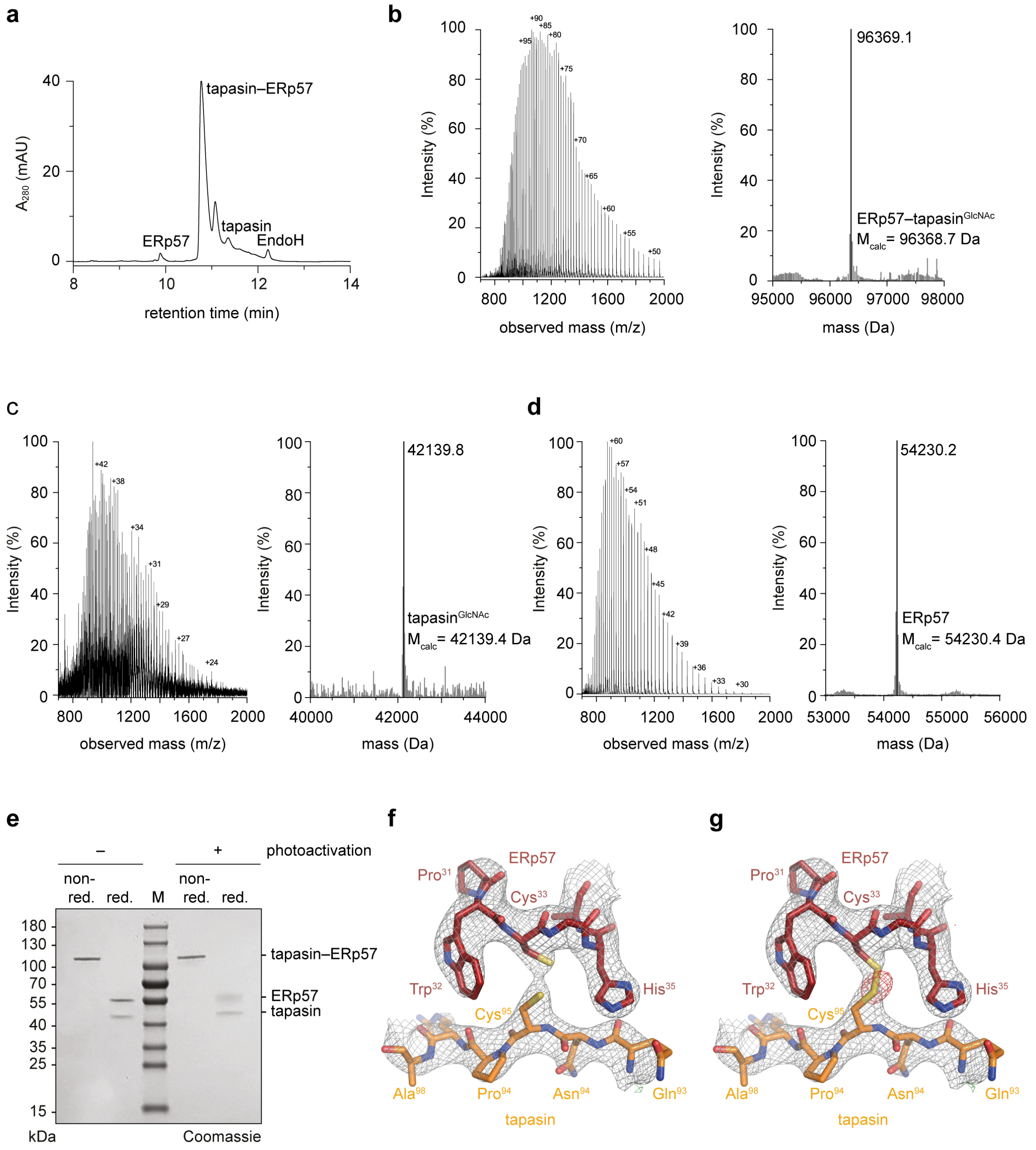

Supplementary Fig. 1 An intact intermolecular disulfide bond is formed in tapasin–ERp57 but is absent in the crystal structure. a The endoH-deglycosylated tapasin–ERp57 complex was analyzed by reversed-phase C_4 chromatography combined with mass spectrometry. A_{280}, absorption at 280 nm. **b** ESI-MS (left) and MaxEnt1-deconvoluted (right) spectrum of corresponding tapasin–ERp57 complex. M_{calc}, calculated mass. **c** ESI-MS (left) and MaxEnt1-

deconvoluted (right) spectrum of tapasin. **d** ESI-MS (left) and MaxEnt1 deconvoluted (right) spectrum of ERp57. **e** SDS-PAGE analysis of the purified tapasin–ERp57 complex under non-reducing (non red.) and reducing (red.) conditions. -/+, samples before and after exposure to UV light; kDa, kilodalton; M, marker; representative SDS-PAGE is shown. **f** Stick representation of residues around Cys^{95} of tapasin (orange) and Cys^{33} of ERp57 (red) with the $2F_o$-F_c map (grey mesh) displayed at the 2 σ level and the F_o-F_c difference map (red mesh) displayed at the -4 σ level. **g** Same representation as in (**f**) but calculated with a modeled disulfide bond between Cys^{95} of tapasin and Cys^{33} of ERp57. Source data for (**a**) and (**e**) are provided as a Source Data file.

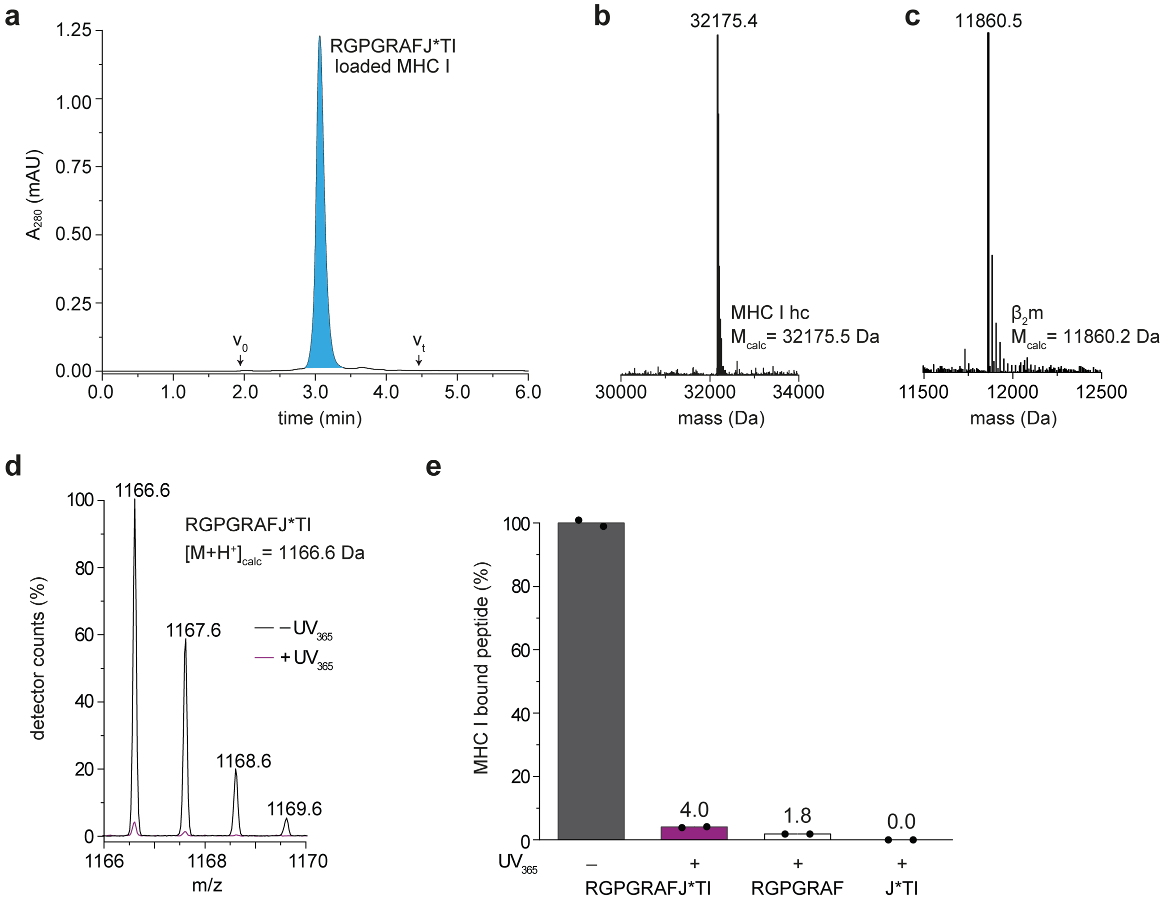

Supplementary Fig. 2 Photo-triggered cleavage of MHC I-associated peptide analyzed by SEC-MS. a Size exclusion chromatogram of RGPGRAFJ*TI loaded MHC I. Combined ESI-MS spectra of the main peak (light blue) were used for MS analysis shown in (**b-e**). The intact pMHC I complex is decomposed into individual components during ionization. A_{280}, absorption at 280 nm; V_0, void volume; V_t, total volume. **b-d** Intact protein masses for MHC I hc (**b**), β$_2$m (**c**) and MHC I-associated RGPGRAFJ*TI peptide (**d**, black line) were detected by ESI-MS. No covalently linked pMHC I complexes were detected after UV illumination at 365 nm. Amounts of MHC I-associated RGPGRAFJ*TI peptides after photo-triggered peptide removal are shown (**d**, purple line). M_{calc}, calculated mass. **e** Quantification of MHC I-associated photocleavable peptides and peptide fragments before (dark grey) and after illumination at 365 nm (intact peptide, purple; RGPGRAF fragment, white) followed by SEC-MS. Quantification is based on MS detector response and normalized to non-photocleaved pMHC I. The short J*TI fragment was not detected. n=2 biological replicates. Source data for (**a**) and (**e**) are provided as a Source Data file.

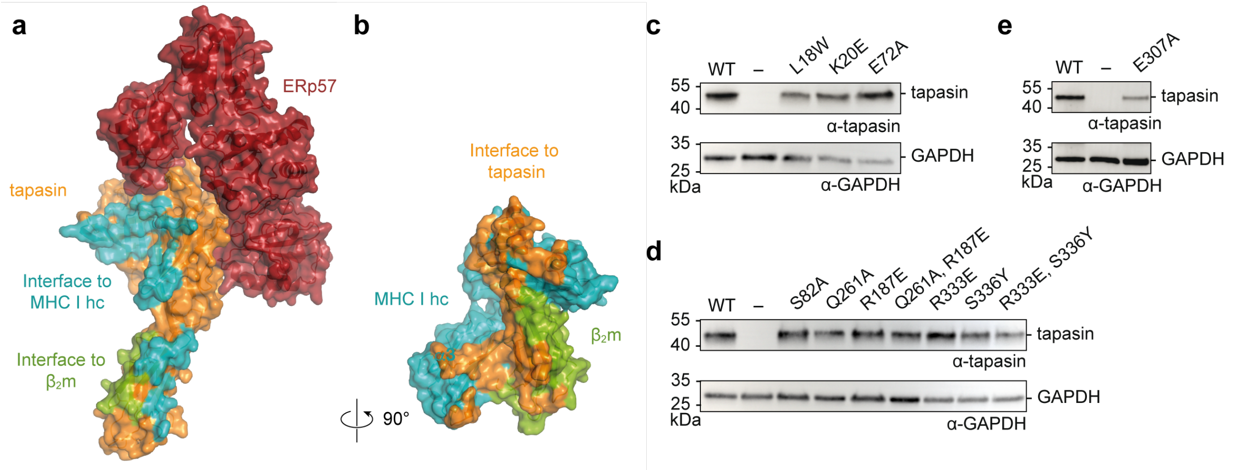

Supplementary Fig. 3 Interaction interfaces in the MHC I–tapasin–ERp57 editing complex. **a** Contact regions (surface representation) of MHC I hc and β_2m on the tapasin–ERp57 heterodimer, colored in orange and green, respectively. hc, heavy chain; β_2m, β_2-microglobulin. **b** Contact regions of tapasin on the MHC I hc and β_2m, colored in orange. **c** Whole cell extracts of eGFP-positive cells, expressing wildtype (WT) or F-pocket interface mutants of tapasin were analyzed by SDS-PAGE and immunoblotting (α-tapasin, α-GAPDH). –, mock transfection; kDa, kilodalton. **d** Immunoblots of whole cell extracts of wildtype and β hairpin, α3-domain interface mutants of tapasin (α-tapasin, α-GAPDH). –, mock transfection. **e** Whole cell extracts of wildtype and β_2m interface mutant of tapasin analyzed by SDS-PAGE and immunoblotting (α-tapasin, α-GAPDH). n=3 biologically independent samples for (**c**), (**d**) and (**e**) –, mock transfection. Source data for (**c**), (**d**), and (**e**) are provided as a Source Data file.

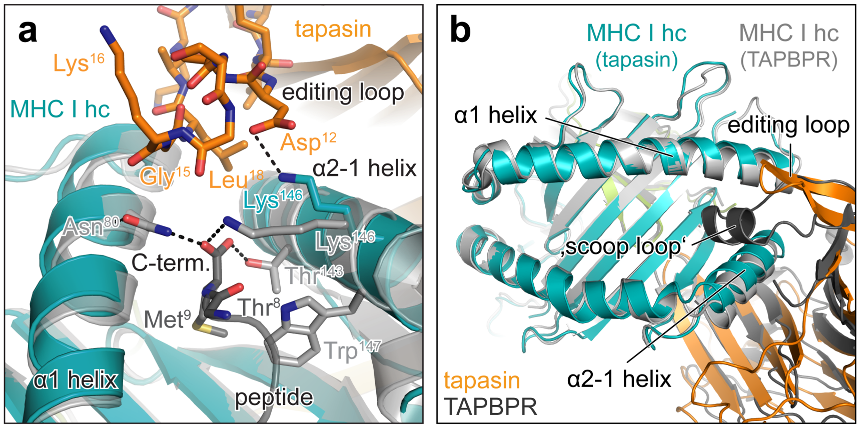

Supplementary Fig. 4 Comparison of peptide-bound with chaperoned MHC I. a View onto the F-pocket region of peptide-bound H2-D^{b} (grey, PDB ID 2F74) with superposition of tapasin (orange)-complexed H2-D^{b} (teal). Hydrogen bonds and the salt bridge of Lys^{146} are indicated by black dashed lines. Only backbone atoms are shown for Thr^{8} of the MHC I-bound peptide. C-term., C terminus. **b** Comparison between the TAPBPR (dark grey)–MHC I (grey) complex (PDB ID 5OPI) and the tapasin (orange)–MHC I (teal) complex in cartoon representation.

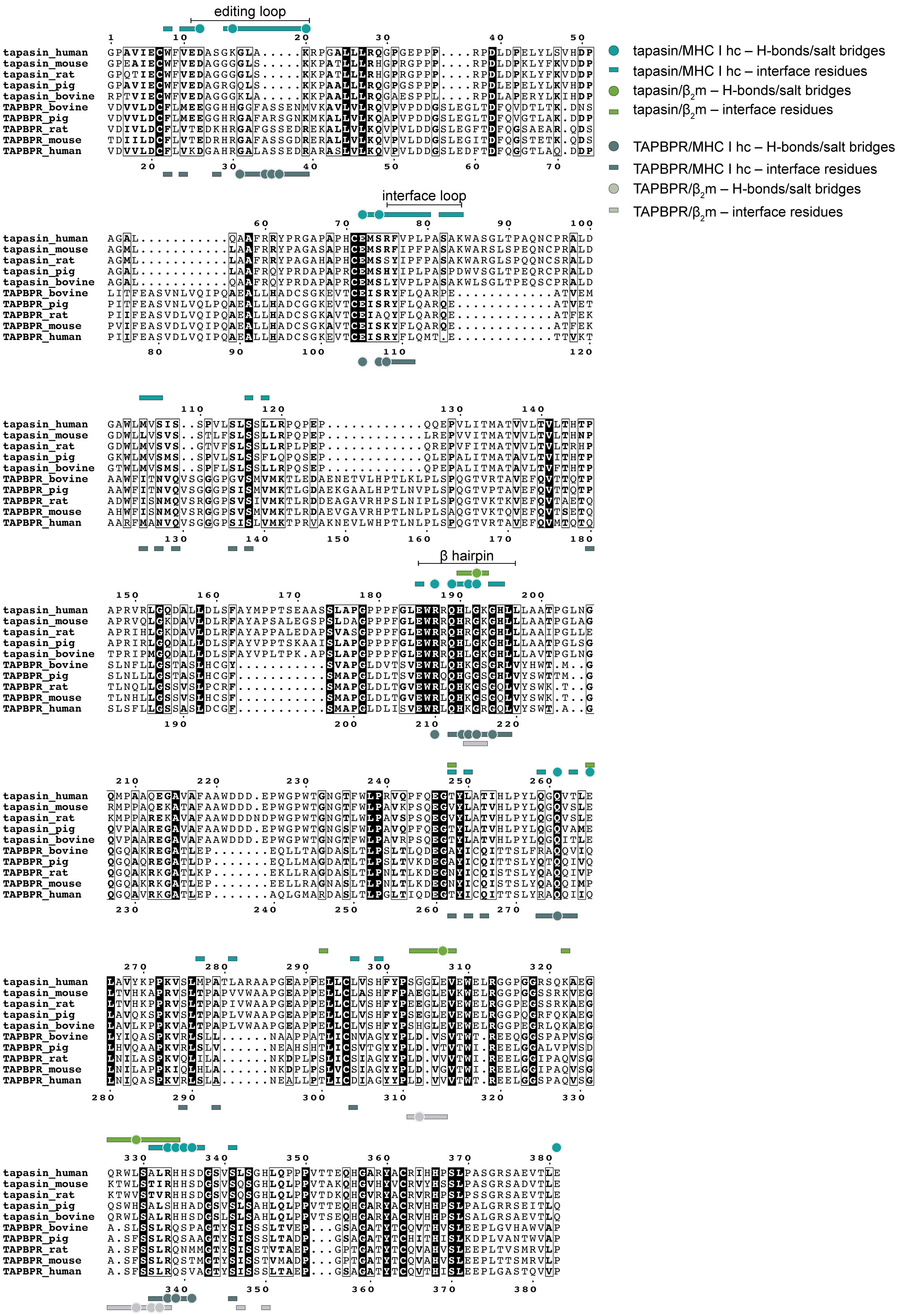
editing loop
interface loop
β hairpin
tapasin/MHC I hc – H-bonds/salt bridges
tapasin/MHC I hc – interface residues
tapasin/β2m – H-bonds/salt bridges
tapasin/β2m – interface residues
TAPBPR/MHC I hc – H-bonds/salt bridges
TAPBPR/MHC I hc – interface residues
TAPBPR/β2m – H-bonds/salt bridges
TAPBPR/β2m – interface residues

Supplementary Fig. 5 Structure-based sequence alignment of tapasin and TAPBPR. Conserved residues are highlighted in black, and physiochemically related residues are represented in bold letters. Residues of tapasin that are involved in interactions with MHC I and β_2m are marked with teal and green dots and bars, respectively. Residues of TAPBPR engaged in interaction with MHC I and β_2m are marked with dark and light symbols, respectively. The dots highlight specific interactions, such as hydrogen bonds and salt bridges. hc, heavy chain; β_2m, β_2-microglobulin.

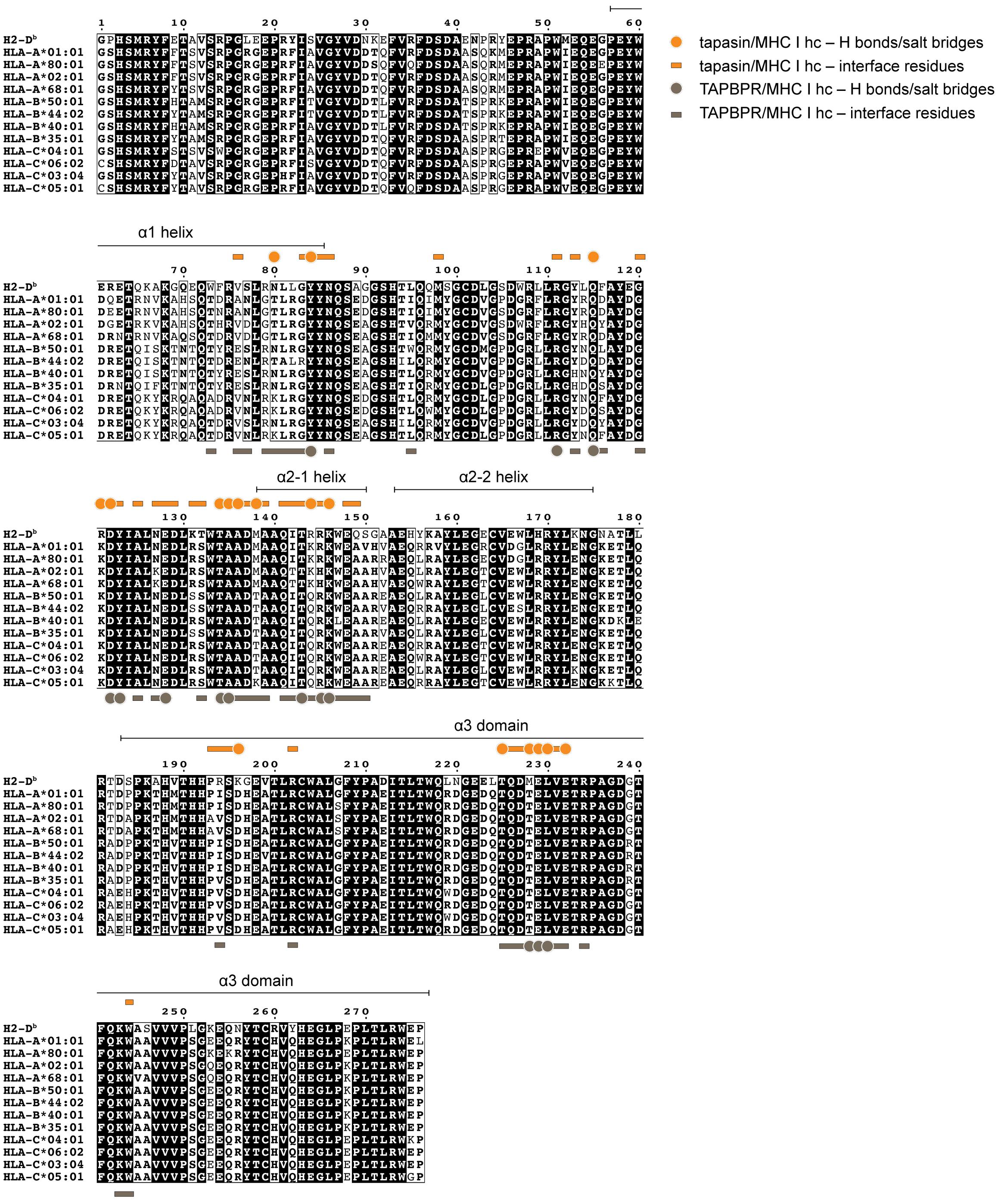

Supplementary Fig. 6 Multiple sequence alignment of classical MHC I heavy chains. Conserved residues are highlighted in black, and physiochemically related residues are represented in bold letters. Orange dots and bars mark residues of MHC I hc that are involved in interactions with tapasin. Residues of MHC I hc engaged in interaction with TAPBPR are marked as for tapasin but with brown symbols. The dots highlight specific interactions, such as hydrogen bonds and salt bridges. hc, heavy chain.

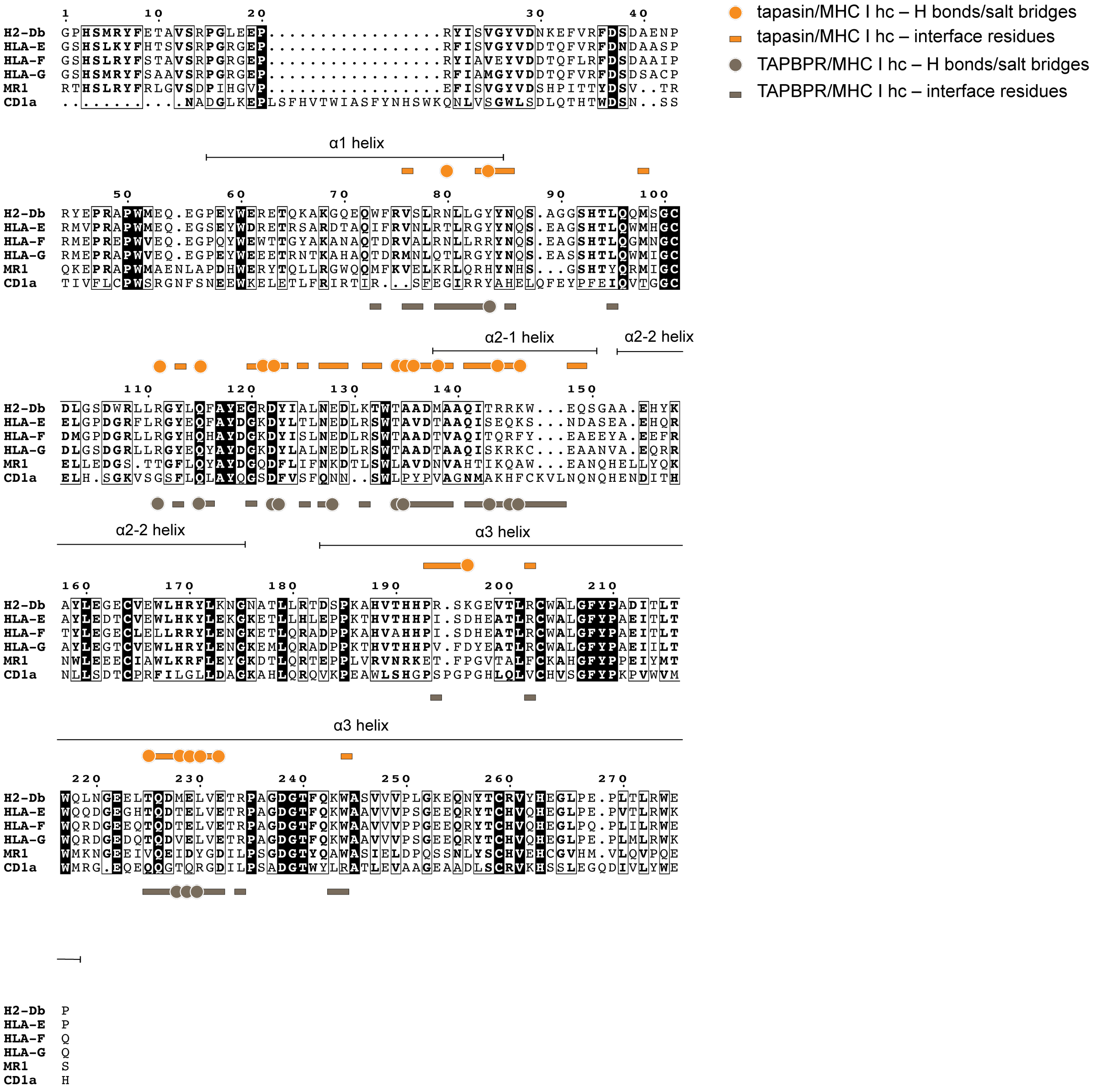

Supplementary Fig. 7 Multiple sequence alignment of non-classical MHC I and related molecules. Conserved residues are highlighted in black and physiochemically related residues are represented in bold letters. Orange dots and bars mark residues of MHC I hc that are involved in interactions with tapasin. Residues of MHC I hc engaged in interaction with TAPBPR are as for tapasin but marked with brown symbols. The dots highlight specific interactions, such as hydrogen bonds and salt bridges. hc, heavy chain.

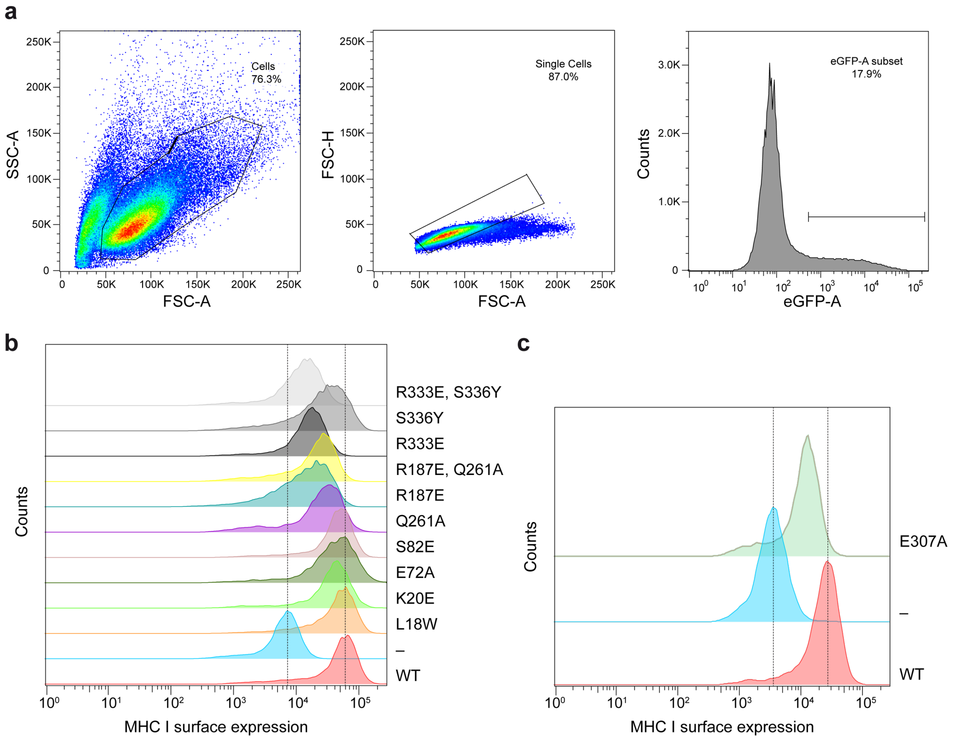

Supplementary Fig. 8 Gating strategy and histograms of flow cytometry analyses. **a** Transfected tapasin-deficient HAP1 cells were first gated based on their size and granularity (SSC-A/FSC-A), duplets were discriminated (FSC-H/FSC-A), and final gate was set based on eGFP fluorescence (histogram). **b** Histogram representation of MHC I surface expression of wildtype (red, WT; mock, blue) and F-pocket (orange, L18W; green, K20E; dark green, E72A), interface loop (purple, Q261A), β hairpin (blue-green, R187E; yellow, R187E, Q261A), α3-domain (dark grey, R333E; grey, S336Y; light grey, R333E, S336Y) interface mutants of tapasin, shown in Fig. 3e and Fig. 4f. **c**, Histogram representation of surface MHC I of wildtype (red, WT; blue, mock) and β_2m interface mutant of tapasin (green, E307A) shown in Fig. 4f. WT, wildtype; –, mock transfection.

SUPPLEMENTARY TABLES

Supplementary Table 1 Data collection and refinement statistics. The structure of MHC I–tapasin–ERp57 complex was determined from one protein crystal. Statistics for the highest-resolution shell are given in parentheses.

	**MHC I–tapasin–ERp57 complex**
Wavelength	1.033
Resolution range (Å)	48.0 - 2.7 (2.8 - 2.7)
Space group	$P22_12_1$
Unit cell dimensions (Å)	a = 74.76, b = 168.53, c = 187.05
Total reflections	388317 (38711)
Unique reflections	65719 (6491)
Multiplicity	5.9 (6.0)
Completeness (%)	99.76 (99.86)
Mean I/sigma(I)	14.90 (1.02)
Wilson B-factor	87.61
R-merge	0.07144 (1.507)
R-meas	0.0784 (1.65)
R-pim	0.03178 (0.6626)
CC1/2	0.998 (0.443)
CC*	1 (0.783)
Reflections used in refinement	65719 (6488)
Reflections used for R-free	3284 (324)
R-work	0.1983 (0.3229)
R-free	0.2289 (0.3445)
Number of non-hydrogen atoms	9726
macromolecules	9635
ligands	39
solvent	52
Protein residues	1214
RMS(bonds)	0.009
RMS(angles)	1.36
Ramachandran favored (%)	95.00
Ramachandran allowed (%)	5.00
Ramachandran outliers (%)	0.00
Rotamer outliers (%)	1.10
Clashscore	5.46
MolProbity score	1.64
Average B-factor	106.17
macromolecules	106.23
ligands	128.14
solvent	79.25
Number of TLS groups	21

Supplementary Table 2 Primers used in this study.

Primer	Sequence (5'-3')
BamHI_Tapasin_ΔTMD_for	CTGTTTGGATCCATGAAGTCCC TGTCTCTGCTC
Tapasin_ΔTMD_His6_HindIII_rev	CTCGACAAGCTTTCATGATCCA TGATGATGATGATGGTGTGATC CCTCCAGGGTGACCTCAG
BamHI_ERp57_C36A_for	TGTTTGGATCCATGCGTCTCCG TCGCCTAGCGC
ERp57_C36A_XbaI_rev	CAGGCTCTAGATTAGAGATCCT CCTGTGCCTTCTTC
Tapasin_ ΔTMD_TEV_His6_SLIC_for	GAAAACCTGTATTTTCAGGGCC ACCATCATCATCATCATGG
Tapasin_ ΔTMD_TEV_His6_SLIC_rev	CAGACTTACCGAGAGtgCCTGC GCAC
Tapasin_NotI_SLIC_for	AAGCTTGTCGCGGCCGCGAGA AGTACTAG
Tapasin_NotI_SLIC_rev	GCGGCCGCGACAAGCTTTCAC TC
NotI_IRES2_eGFP_for	CGCGCGGCCGCCCCCTCTCCC TCCCCCCCCCCTAAC
IRES2_eGFP_ScaI_rev	CGCAGTACTTTACTTGTACAGC TCGTCCATGCCG
Tapasin_L18W_SLIC_for	GAAAGGGCTGGGCCAAGAGAC CCGGTG
Tapasin_L18W_SLIC_rev	CTTGGCCCAGCCCTTTCCGCTC GC
Tapasin_K20E_SLIC_for	CCTGGCCGAGAGACCCGGTGC AC
Tapasin_K20E_SLIC_rev	GGGTCTCTCGGCCAGGCCCTT TCC
Tapasin_E72A_SLIC_for	CCACACTGCGCGATGAGCCGC TTCG
Tapasin_E72A_SLIC_rev	CTCATCGCGCAGTGTGGTGCG GG
Tapasin_Q261A_SLIC_for	GCAAGGAGCGGTCACCCTGGA GCTTG
Tapasin_Q261A_SLIC_rev	GGGTGACCGCTCCTTGCAGGT ATGGC
Tapasin_R187E_SLIC_for	CTAGAGTGGGAACGCCAGCAC CTGGG
Tapasin_R187E_SLIC_rev	GTGCTGGCGTTCCCACTCTAGC CCAAAG
Tapasin_R333E_SLIC_for	CGGCCCTGGAACACCATTCCG ATGGCTC
Tapasin_R333E_SLIC_rev	GAATGGTGTTCCAGGGCCGAG AGCCACC

Tapasin_S336Y_SLIC_for	GCGCCACCATTACGATGGCTCT GTCAGC
Tapasin_S336Y_SLIC_rev	CATCGTAATGGTGGCGCAGGG CCG
Tapasin_E307A_SLIC_for	CAGGGGCGGCACCCCCGG
Tapasin_E307A_SLIC_rev	GGTGCCGCCCCTGGGGCG
