# Supplementary material for: Structure of an MHC I–tapasin–ERp57 editing complex defines chaperone promiscuity
Source: Nat Commun. 2022 Sep 14;13:5383. doi: 10.1038/s41467-022-32841-9 (PMC9474470; doi:10.1038/s41467-022-32841-9)
Supplement: Supplementary file 3 — Description of Additional Supplementary Files [file 41467_2022_32841_MOESM3_ESM.pdf]

File name: Supplementary Movie 1

Description: Crystal structure of an MHC I–tapasin– ERp57 chaperone complex at 2.7 Å resolution. Important features of MHC I interaction, peptide proofreading, and loading are displayed. The editor loop of tapasin, which contributes to a widened F-pocket of peptidereceptive MHC I is shown with its electron density (contour level: 1.5 σ).
