# Supplementary material for: Structure of an MHC I–tapasin–ERp57 editing complex defines chaperone promiscuity
Source: Nat Commun. 2022 Sep 14;13:5383. doi: 10.1038/s41467-022-32841-9 (PMC9474470; doi:10.1038/s41467-022-32841-9)
Supplement: Supplementary file 5 — Reporting Summary [file 41467_2022_32841_MOESM5_ESM.pdf]

nature portfolio

Corresponding author(s): Simon Trowitzsch, Robert Tampé

Last updated by author(s): Aug 9, 2022

Reporting Summary

Nature Portfolio wishes to improve the reproducibility of the work that we publish. This form provides structure for consistency and transparency in reporting. For further information on Nature Portfolio policies, see our Editorial Policies and the Editorial Policy Checklist.

Statistics

For all statistical analyses, confirm that the following items are present in the figure legend, table legend, main text, or Methods section.

n/a	Confirmed	
☐	☒	The exact sample size (*n*) for each experimental group/condition, given as a discrete number and unit of measurement
☐	☒	A statement on whether measurements were taken from distinct samples or whether the same sample was measured repeatedly
☒	☐	The statistical test(s) used AND whether they are one- or two-sided<br>*Only common tests should be described solely by name; describe more complex techniques in the Methods section.*
☒	☐	A description of all covariates tested
☒	☐	A description of any assumptions or corrections, such as tests of normality and adjustment for multiple comparisons
☐	☒	A full description of the statistical parameters including central tendency (e.g. means) or other basic estimates (e.g. regression coefficient) AND variation (e.g. standard deviation) or associated estimates of uncertainty (e.g. confidence intervals)
☒	☐	For null hypothesis testing, the test statistic (e.g. *F*, *t*, *r*) with confidence intervals, effect sizes, degrees of freedom and *P* value noted<br>*Give P values as exact values whenever suitable.*
☒	☐	For Bayesian analysis, information on the choice of priors and Markov chain Monte Carlo settings
☒	☐	For hierarchical and complex designs, identification of the appropriate level for tests and full reporting of outcomes
☒	☐	Estimates of effect sizes (e.g. Cohen's *d*, Pearson's *r*), indicating how they were calculated

Our web collection on statistics for biologists contains articles on many of the points above.

Software and code

Policy information about availability of computer code

Data collection	X-ray crystallography: CBFlip v0.7.8 (Dectris, Crystallography Binary File (CBF) version 1.5)<br>Mass spectrometry: Unify 1.9.4 (Waters Corporation)<br>Flow cytometry: BD FACSChorus 1.1.19.0
Data analysis	X-ray crystallography: XDS (built 20200131), Coot (Version 0.9.3), Phenix (version 1.19.2_4158), MacPyMOL (v1.7.2.0), PDB-REDO server (REFMAC 5.8.0267)<br>Mass spectrometry: Unify 1.9.4 (Waters Corporation)<br>FlowCytometry: FlowJo V10, Excel (Version 16.7) Prism 8.21 MacOS (Graphpad Software)<br>Sequence alignments: Clustal Omega 0(1.2.4), ESPript 3.0

For manuscripts utilizing custom algorithms or software that are central to the research but not yet described in published literature, software must be made available to editors and reviewers. We strongly encourage code deposition in a community repository (e.g. GitHub). See the Nature Portfolio guidelines for submitting code & software for further information.

Data

Policy information about availability of data

All manuscripts must include a data availability statement. This statement should provide the following information, where applicable:

- Accession codes, unique identifiers, or web links for publicly available datasets
- A description of any restrictions on data availability
- For clinical datasets or third party data, please ensure that the statement adheres to our policy

X-ray crystallography: Atomic coordinates and structure factors were deposited to the Protein Data Bank (PDB ID 7QNG). Previously published structural data used

in this study are, as stated in the manuscript, accessible at the PDB (peptide-receptive MHC I, PBD ID 2F74; client-free tapasin-ERp57 heterodimer, PDB ID 2F8U; MHC I-TAPBPR complex, PDB ID 5OPI).
Mass spectrometry: Data is available at Zenodo, DOI: 10.5281/zenodo.5939241.
Other data are included in the source data file.

Field-specific reporting

Please select the one below that is the best fit for your research. If you are not sure, read the appropriate sections before making your selection.

☒ Life sciences ☐ Behavioural & social sciences ☐ Ecological, evolutionary & environmental sciences

For a reference copy of the document with all sections, see nature.com/documents/nr-reporting-summary-flat.pdf

Life sciences study design

All studies must disclose on these points even when the disclosure is negative.

Sample size	No statistical methods were applied to determine sample size.<br>Flow cytometry experiments were carried out at least twice (biological replica).<br>Also, Western Blots of whole cell extracts and SEC-MS were performed at least twice (WB, three biological replica; SEC-MS, two biological replica).
Data exclusions	No data were excluded from analyses.
Replication	All replications were successful. For flow cytometry, Western Blots and SEC-MS, all experiments were replicated at least twice. Number of replica are stated in each figure legend.
Randomization	Randomization was not relevant to this study, because no research on living organisms was performed, thus no confounding factors, which require randomization, were expected.
Blinding	Blinding was not relevant to any experiments present in this study. Since no research on living organisms was included, no confounding factors, requiring blinding were expected.

Reporting for specific materials, systems and methods

We require information from authors about some types of materials, experimental systems and methods used in many studies. Here, indicate whether each material, system or method listed is relevant to your study. If you are not sure if a list item applies to your research, read the appropriate section before selecting a response.

Materials & experimental systems

n/a	Involved in the study	
☐	☒	Antibodies
☐	☒	Eukaryotic cell lines
☒	☐	Palaeontology and archaeology
☒	☐	Animals and other organisms
☒	☐	Human research participants
☒	☐	Clinical data
☒	☐	Dual use research of concern

Methods

n/a	Involved in the study	
☒	☐	ChIP-seq
☐	☒	Flow cytometry
☒	☐	MRI-based neuroimaging

Antibodies

Antibodies used	Flow cytometry: Anti-HLA-A/B/C antibody, mouse IgG2a,κ, labeled with APC, BioLegend (clone W6/32, Cat #311409)<br>Immunoblotting: Anti-tapasin antibody, hybridoma supernatant, generated in-house (clone 7F6); anti-GAPDH antibody, monoclonal mouse, HRP conjugated, BioLegend (clone FF26A/F9, Cat #649203); anti-GAPDH antibody, polyclonal rabbit, unconjugated, Thermo Fisher Scientific (Cat #PA-987), anti-rat IgG Peroxidase conjugate antibody, polyclonal goat, Sigma Aldrich (Cat #a9037); anti-rabbit IgG Peroxidase conjuagte antibody, polyclonal goat, Merck Millipore (Cat #AP307P).
Validation	Anti-HLA-A/B/C antibody is validated for flow cytometry of human MHC class I by the manufacturer as stated on the manufacturer's website, corresponding product publication by Madrid et al., J Virol. 86, 8693-8704, 2012.<br>Anti-tapasin antibody, hybridoma supernatant, generated in-house (clone 7F6) see Hulpke et al., Cell. Mol. Life Sci. 69, 3317-3327, 2012, validated for immunoblotting of human tapasin.<br>HRP-conjugated anti-GAPDH antibody is validated for immunoblotting of human GAPDH by the manufacturer as stated on the manufacturer's website, corresponding product publication by Wang et al., Sci Rep. 9, 1853, 2019.<br>Unconjugated anti-GAPDH antibody is validated for immunoblotting of human GAPDH by the manufacturer as stated on the manufacturer's website, corresponding product publication by Zhao et al., Nat Commun. 12, 3691, 2021.

Secondary antibodies, anti-rat and anti-rabbit, have been validated for immunoblotting by the manufacturers as stated on manufacturers website.

Eukaryotic cell lines

Policy information about cell lines

Cell line source(s)	Spodoptera frugiperda 21 cell line, Invitrogen (11497013), HAP1Δtapasin cell line provided by Robbert Spaapen, Sanquin Research, University of Amsterdam, Netherlands (Jongsma et al., Immunity 54, 132-150 e139, 2021).
Authentication	The targeted gene region of the HAP1Δtapasin cell line was confirmed by Sanger sequencing and absence of tapasin was validated by immunoblotting (Jongsma et al., Immunity 54, 132-150 e139, 2021).<br>Morphology of Spodoptera frugiperda 21 cells were investigated when cells were cultured.
Mycoplasma contamination	Spodoptera frugiperda 21 cell line was not tested for mycroplasma constmination.<br>HAP1Δtapasin cell line was regularly tested negativ for microplasma contamination.
Commonly misidentified lines<br>(See ICLAC register)	No commonly misidentified lines were used in this study.

Flow Cytometry

Plots

Confirm that:

☒ The axis labels state the marker and fluorochrome used (e.g. CD4-FITC).

☒ The axis scales are clearly visible. Include numbers along axes only for bottom left plot of group (a 'group' is an analysis of identical markers).

☒ All plots are contour plots with outliers or pseudocolor plots.

☒ A numerical value for number of cells or percentage (with statistics) is provided.

Methodology

Sample preparation	Cells were washed with 1x DPBS (Gibco), detached using 0.05% Trypsin-EDTA (Gibco) and washed with FACS buffer (2% BSA, 2mM EDTA, 0.02% sodium azide in DPBS). Then, cells were blocked with 10% FCR blocking reagent, human (Miltenyi Biotec), washed with FACS buffer and stained with 2 µl of APC anti-human HLA-A/B/C antibody (BioLegend) for 30 min, washed with FACS buffer and resuspended in FACS buffer for analysis.
Instrument	FACSMelody Cell Sorter (BD Bioscience)
Software	FlowJo V10 software, BD FACSChorus 1.1.19.0
Cell population abundance	Abundance of construct-expressing cells was determined by gating on eGFP positive cells (FlowJo V10 software). 17-51% of parental gate cells were eGFP expressing cells.
Gating strategy	For analysis of MHC class I, gating on cell population was based on size and granularity (SSC-A/FSC-A), duplets were discriminated (FSC-H/FSC-A) and construct-expressing cells were gated based on eGFP fluorescence (histogramme).

☒ Tick this box to confirm that a figure exemplifying the gating strategy is provided in the Supplementary Information.
